# Comprehensive Assessment of Serum hsa_circ_0070354 as a Novel Diagnostic and Predictive Biomarker in Non-small Cell Lung Cancer

**DOI:** 10.3389/fgene.2021.796776

**Published:** 2022-01-13

**Authors:** Yuejiao Huang, Shiyi Qin, Xinliang Gu, Ming Zheng, Qi Zhang, Yupeng Liu, Chun Cheng, Kaibin Huang, Chunlei Peng, Shaoqing Ju

**Affiliations:** ^1^ Department of Medical Oncology, Affiliated Hospital of Nantong University, Nantong, China; ^2^ Medical School of Nantong University, Nantong, China; ^3^ Department of Laboratory Medicine, Affiliated Hospital of Nantong University, Nantong, China; ^4^ Department of Thoracic Surgery, Affiliated Tumor Hospital of Nantong University, Nantong, China; ^5^ Department of General Surgery, Nantong Haimen People’s Hospital, Nantong, China; ^6^ Department of Medical Oncology, Affiliated Tumor Hospital of Nantong University, Nantong, China

**Keywords:** circular RNA, non-small cell lung cancer, diagnosis, prognosis, biomarker

## Abstract

**Background:** More and more studies have shown that circular RNAs (circRNAs) play an essential role in the occurrence and development of tumors. Hence, they can be used as biomarkers to assist in diagnosing tumors. This study focuses on exploring the role of circular RNA (hsa_circ_0070354) in the diagnosis and prognosis of non-small cell lung cancer (NSCLC).

**Materials and Methods:** First of all, high-throughput sequencing was used to find the difference in the expression of circular RNA between NSCLC and adjacent tissues. The circRNAs with higher differences in expression were selected to verify their expressions in tissues, cells, and serum using qRT-PCR. Secondly, the hsa_circ_0070354 with a significant difference was chosen as the research goal, and the molecular properties were verified by agarose gel electrophoresis and Sanger sequencing, etc. Then, actinomycin D and repeated freeze-thaw were used to explore the stability and repeatability of hsa_circ_0070354. Finally, the expression of hsa_circ_0070354 in serum of 133 patients with NSCLC and 97 normal donors was detected, and its sensitivity, specificity, and prognosis as tumor markers were statistically analyzed.

**Results:** Hsa_circ_0070354 was highly expressed in tissues, cells, and serum of NSCLC, and it has the characteristics of sensitivity, stability, and repeatability. The ROC curve indicates that hsa_circ_0070354 is superior to conventional tumor markers in detecting NSCLC, and the combined diagnosis is of more significance in the diagnosis. The high expression of hsa_circ_0070354 is closely related to the late-stage, poor differentiation of the tumor and the short survival time of the patients, which is an independent indicator of poor prognosis.

**Conclusion:** Hsa_circ_0070354 is not only a novel sensitive index for the diagnosis of NSCLC but also a crucial marker for bad biological behavior.

## Introduction

Previously, lung cancer has been the leading malignant tumor with the highest morbidity and mortality globally. Although the global cancer data in 2020 showed that the incidence rate of lung cancer has changed from the first place, the mortality rate was still the highest (Ma et al., 2021). In Asia, the incidence and mortality of lung cancer occupy the first place in the global data, and China is at the top of the list compared to other countries. Even though risk factors such as smoking are under control, the incidence rises in both sexes (Zukotynski et al., 2021). In all lung cancer cases, non-small cell lung cancer (NSCLC) accounts for more than 80%, with a 5-year survival rate of only about 15% (Ferlay et al., 2010; Hang et al., 2018) and a recurrence rate after radical treatment of more than 40% (Zheng et al., 2013). In the past 2 decades, an endless stream of treatments has been widely applied to NSCLC to reduce mortality and improve quality of life. Compared with conventional radiotherapy and chemotherapy, molecular targeting and immunotherapy have been preferred for patients with advanced, recurrent, and metastatic NSCLC (Ma et al., 2021). However, radical surgery for early NSCLC is still the key to curing the disease (Chaft et al., 2021). The reason for the high mortality rate of NSCLC is that patients were diagnosed at an advanced stage or local late-stage, missing the possibility of early cure. The leading causes include lack of routine physical examination, lack of biomarkers for early diagnosis, and disease surveillance methods (Qian et al., 2021). Therefore, with the progress of imaging, more sensitive and specific tumor biomarkers are needed to screen and diagnose NSCLC.

Circular RNAs (CircRNAs) are a class of non-coding RNAs derived from single or multiple exons (ENCODE Project Consortium, 2012; Zhao et al., 2019), a unique subtype of non-coding RNAs, with a covalently, closed continuous loop structure and no polarized or polyadenylated tail (Memczak et al., 2013; Rybak-Wolf et al., 2015). Unlike the traditional linear RNAs, circRNA has a high tolerance to exonuclease and is widely and stably expressed in the cytoplasm of various eukaryotic cells (Glažar et al., 2014; Zhao et al., 2019; Pei et al., 2020).

CircRNAs have always been regarded as by-products of low abundance splicing or splicing errors (Nigro et al., 1991; Cocquerelle et al., 1993). With the rapid development of high-throughput sequencing and bioinformatics technology, plenty of circRNAs have sprung up and been studied (Jeck et al., 2013; Memczak et al., 2013; Jeck and Sharpless, 2014). CircRNAs are generally considered the primary regulators of cellular processes because of their unique properties, such as richness, stability, and specific expression in cells and tissues (Di et al., 2019; Yu et al., 2020). CircRNAs can regulate gene expression at the transcriptional and post-transcriptional level by interacting with microRNAs (miRNAs) or other competing endogenous RNAs and participate in various biological activities (Hansen et al., 2013; Peng et al., 2015; Wang et al., 2018). Growing evidence showed that abnormal expression of circRNA may lead to the occurrence and development of cancers, including NSCLC (Wan et al., 2016; Zhu et al., 2017). Besides, numerous researches have identified that circRNAs are related to the clinical characteristics of patients and play a regulatory role in NSCLC (Zhang et al., 2019).

Tumor biomarkers are essential methods for tumor screening and early diagnosis due to their multiple sampling, dynamic monitoring, economic efficiency, as well as the advantages of high sensitivity and specificity. At present, the most commonly used serum tumor biomarkers for the diagnosis of NSCLC are embryonic antigen (CEA), squamous cell carcinoma antigen (SCC), and cytokeratin 19 fragment (Cyfra21-1) (Ye et al., 2020). However, the sensitivity and specificity of the above three markers for diagnosing NSCLC are not satisfactory, especially in the early stage of the disease (Kamel et al., 2019). CircRNAs play a vital role in the occurrence and development of NSCLC and can stably exist in patients’ body fluids. Combined with their high sensitivity and specificity, they are ideal choices for liquid biopsy. Therefore, circRNAs can be considered as new specific diagnostic biomarkers of NSCLC (Huang et al., 2021a). Existing studies have successively verified the feasibility of circRNAs as a biomarker for the early diagnosis of NSCLC. For example, the differential expression of circ_0047921, circ_0056285, and circ_0007761 in exosomes can distinguish early NSCLC from healthy people, chronic obstructive pulmonary disease (COPD), or pulmonary tuberculosis (Xian et al., 2020). The high expression of hsa_circ_0102533 in peripheral blood indicated the occurrence of NSCLC (Zhou et al., 2018). Nevertheless, hsa_circ_0046264 was highly expressed in serum of patients with NSCLC, and it was closely related to clinical factors such as the age of onset, tumor size, disease stage, and lymph node metastasis (Liu et al., 2020). Moreover, circRNAs can also be regarded as predictors of adverse biological behaviors of lung cancer. For instance, a statistical difference between the high expression of hsa_circ_0060937 in serum and the occurrence of bone metastasis occurred in NSCLC (Zhang et al., 2020). The increased expression of circFARSA in plasma of patients was closely related to the migration and invasion of tumor cells as well (Hang et al., 2018). What’s more, circRNAs could be used as biological indicators to guide treatment. Specifically, the high expression of circPVT1 often indicates that patients are not sensitive to chemotherapy (Lu et al., 2021). Likewise, the expressions of hsa_circ_0134501 and hsa_circ_0109320 were low in NSCLCs’ plasma, and the combination of them can be used as a predictor of response to gefitinib (Liu et al., 2019a). Similarly, in the immunotherapy of NSCLC, circ-CPA4 participated in the regulation of CD8^+^T cell response (Hong et al., 2020), while the overexpression of circFGFR1 led to drug resistance to programmed cell death protein-1 (PD-1) (Zhang et al., 2019).

In this study, through high-throughput sequencing, it was found that a dramatic difference in the expression of hsa_circ_0070354 between NSCLC and adjacent tissues. Our team demonstrated the expression of hsa_circ_0070354 in serum of patients with NSCLC for the first time, analyzed its correlation with clinical features, evaluated its diagnostic efficacy, and explored its possibility as a novel biomarker for diagnosing NSCLC.

## Materials and Methods

### Serum and Tissue Samples

The serum samples of 133 patients with NSCLC (I-IV stage) and 97 healthy donors were collected from the affiliated Hospital of Nantong University from 2015 to 2016 for follow-up experimental analysis. The sera collected by us were those after the initial diagnosis without any treatment. The preoperative and postoperative sera were the samples within 1 week before and after the operation. A flowchart showing the inclusion/exclusion criteria and analysis pipeline of this investigation were provided in Supplementary Figure S1. The tissue samples of NSCLC came from the thoracic surgery of the Affiliated Hospital of Nantong University and the Affiliated Tumor Hospital of Nantong University. Special thanks to Yupeng Liu, Guanjun Ju, Chun Cheng, and others of thoracic surgery for their strict control over the quality inspection of tissue samples, collecting tumor tissues, and paracancerous tissues (>5 cm tumor margin) with a diameter greater than 0.5 cm. More than two pathologists diagnosed all the samples, and TNM staging was performed according to the 8th edition of WHO. Every patient signed the informed consent form for collecting and preserving specimens and documenting details. All the samples were immediately kept in enzyme-free aseptic cryopreservation tubes at −80°C for a long time.

### High Throughput Sequencing

Relying on Hipure Total RNA Minikit (Magen, Guangzhou, China), we extracted total RNA from 3 pairs of cancer and paracancerous tissues. Then, the concentration and purity of the total RNAs were evaluated by Qubit 3.0 Fluorometer (Invitrogen, California, CA, United States) and Agilent 2100 Bioanalyzer (Applied Biosystems, California, CA, United States). Ribosomal RNAs were fractionated from total RNA samples, meanwhile linear RNAs were removed (Geneseed, Guangzhou, China), and RNA-seq libraries were orderly constructed by TruSeq RNA sample preparation kits (Illumina, California, CA, United States). The purified cDNA library was sequenced on the Illumina HiSeq Xten platform via the PE150 sequencing model.

### Cell Culture

Four human NSCLC cell lines, A549, H1299, HUT226, SPCA, and normal bronchial epithelial cell line HBE1 were purchased from Shanghai Institutes for Biological Sciences, China Academy of Science (Shanghai, China) and conserved in our laboratory. All cells were cultured in RPMI 1640 medium (Corning, Virginia, VA, United States) containing 10% fetal bovine serum (FBS, Gibco, South Dakota, SD, United States), 100 U/ml penicillin-streptomycin mixture (Gibco, South Dakota, SD, United StatesA) in a humidified incubator (Thermo, Massachusetts, MA, United States) with 5% CO_2_ at 37°C.

### Total RNA Extraction and qRT-PCR

Serum, tissue, and cell total RNAs were extracted by TRIzol reagent (Invitrogen, Karlsruhe, Germany). According to the manufacturer’s instruction, complementary DNAs (cDNAs) were reverse transcribed by Revert Aid RT Reverse Transcription Kit (Thermo Fisher Scientific, Massachusetts, MA, United States). The specific divergent primers involved in this paper were designed and synthesized by Ribobio Corporation (Suzhou, China), with the forward and reverse primer as shown in Table 1. RT-qPCR was performed as described in previous studies (Huang et al., 2021b). In brief, the reaction was initiated at 95°C (10 min), followed by 40 cycles at 95°C (10s) and 62°C (34s) in the 20 ul system. PCR products were identified by Sanger sequencing.

**TABLE 1:** Primer sequences for Real-Time qPCR.

Gene	Primer sequences
has_circ_0070354	Forward: 5′-TCT​GGG​AAA​TCT​TTC​TGG​GTA​A-3′
	Reverse: 5′- CTA​CCA​GAT​GGC​AGC​AAC​AG-3′
PTPN13	Forward: 5′-TTG​CTG​CCA​TCT​GGT​AGT​GTG-3′
	Reverse: 5′-TGG​TGC​AGT​GAA​TGC​TCG​AAG-3′
GAPDH	Forward: 5′-AGA​AGG​CTG​GGG​CTC​ATT​TG-3′
	Reverse: 5′-GCA​GGA​GGC​ATT​GCT​GAT​GAT-3′
U6	Forward: 5′-GAC​TAT​CAT​ATG​CTT​ACC​GT-3′
	Reverse: 5′-GGG​CAG​GAA​GAG​GGC​CTA​T-3′
18S	Forward: 5′-AGA​AGG​CTG​GGG​CTC​ATT​TG-3′
	Reverse: 5′-GCA​GGA​GGC​ATT​GCT​GAT​GAT-3′

### RNase R and Agarose Gel Electrophoresis

RNase R treatment was executed at 37°C with 4 U/μg of RNase R (Epicentre Biotechnologies, Wisconsin, WI, United States) for 3 min and then inactivated at 70°C for 10 min to deactivate the enzyme, after which the mixture was reversely transcribed into cDNA for qRT-PCR. 2.5% agarose gel was prepared, and then the electrophoresis bands of PCR products were observed under UV conditions after about 20 min electrophoretic at 100 V.

### Actinomycin D

The actinomycin D of 1,000 mg/ml was diluted to 2.5 μg/ml using RPMI-1640 complete medium and replaced the ordinary medium for 24 h. After treatment, total RNA was extracted from the cells for subsequent determination at 0, 2, 4, 8, 12, 24 h, respectively.

### Nuclear and Cytoplasmic RNA Separation Assay

Up to 5 × 10^6^ cells were digested by trypsin and collected in a small centrifuge tube for backup use. According to the manufacturer’s protocol, RNA fractions from the nucleus and cytoplasm of cells were separated by the Ambion®PARIS™Kit (Thermo Fisher Scientific, Massachusetts, MA, United States). Nuclear and cytoplasmic RNAs were then converted to cDNAs and detected by qRT-PCR or stored in the refrigerator at −80°C.

### Statistical Analysis

Statistical analyses in this study were mainly conducted by SPSS 20.0 (IBM SPSS Statistics, Chicago, USA) and GraphPad Prism v8.0 (Graphpad Software Inc., California, USA). The differences between groups were analyzed using Student’s t-test and one-way ANOVA. The correlation of hsa_circ_0070354 to clinical characteristics was analyzed by chi-square test. The receiver operating characteristic (ROC) curve and area under the curve (AUC) were determined to evaluate the diagnostic performance of hsa_circ_0070354 in NSCLC. Before plotting the ROC curve, we performed binomial logistic regression. Univariate and multivariate Cox proportional hazards regression models were carried out to assess the clinical variables’ impacts on the overall survival (OS) of NSCLC patients. Survival curves were estimated by the Kaplan-Meier method, while the log-rank test evaluated statistical significance. Data are denoted by mean value ± standard deviation (SD) from at least three biological replicates independently. *p* values < 0.05 were indicated statistical significant.

## Results

### Screening and Detecting the Expression of hsa_circ_0070354 in NSCLC Tissues, Cells, and Sera

**FIGURE 1:**
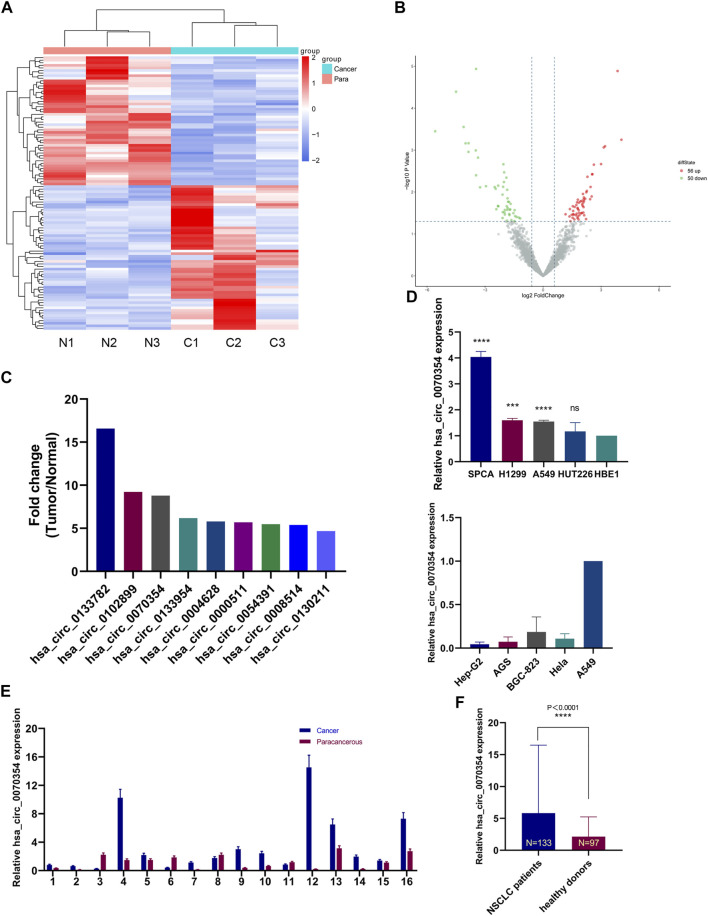
circRNAs microarray sequencing of lung cancer tissues and adjacent tissues. **(A)** The clustered heatmap showed the differentially expressed circRNAs in 3 pairs of human NSCLC tissues and adjacent normal tissues. **(B)** The volcano plots of circRNAs expressions. The red and green strips indicate up-regulated and down-regulated circRNAs, respectively. **(C)** The nine predominantly up-regulated circRNAs between the two groups were shown (>2.0-fold change, *p* < 0.05). **(D–F)** The expression of hsa_circ_0070354 in NSCLC tissues, cells, and serums. Paracancerous tissues, normal lung epithelial cells (HBE1), and healthy donor sera served as controls.

High-throughput sequencing was implemented in 3 pairs of cancerous and adjacent noncancerous tissues from NSCLC patients to determine and characterize the differential expression of circRNAs in NSCLC. A total of 2319 circRNAs were identified, of which 56 were significantly up-regulated and 50 were down-regulated when we set the filter criteria as fold-change ≥ 2 and *p* < 0.05 (Figures 1A,B). Figure 1C showed the top 9 up-regulated circRNAs with remarkable differences, among which our study object hsa_circ_0070354 is included. Then the expression of hsa_circ_0070354 in 16 pairs of NSCLC tissue samples was verified by qRT-PCR (Figure 1E, small sample verification). Notably, 11 cases of adenocarcinoma and 5 cases of squamous cell carcinoma were concluded among the collected cases of NSCLC (Supplementary Table S1). Compared with the corresponding adjacent tissue adjacent tissues, hsa_circ_0070354 showed high expression in tumors. This result also corresponded to the presentation of cell lines. Similarly, compared with normal bronchial epithelial cells, the expression of hsa_circ_0070354 was also increased meaningfully in NSCLC cell lines (Figure 1D above), which indicated that hsa_circ_0070354 might be related to the presence of lung cancer. At the same time, we also detected the expression levels of hsa_circ_0070354 in cell lines of gastric cancer (AGS and BGC-823), hepatocellular carcinoma (Hep-G2), cervical cancer (Hela), and NSCLC (A549), respectively (Figure 4D below). Compared with A549, their CT value was significantly higher (Figure 1D below). Under the verification of large numbers of serum samples, the expression of hsa_circ_0070354 was detected in the serum of 133 patients with NSCLC and 97 normal donors and verified the conclusion of its high expression (Figure 1F).

### Molecular Properties and Methodological Evaluation of hsa_circ_0070354 in NSCLC

**FIGURE 2:**
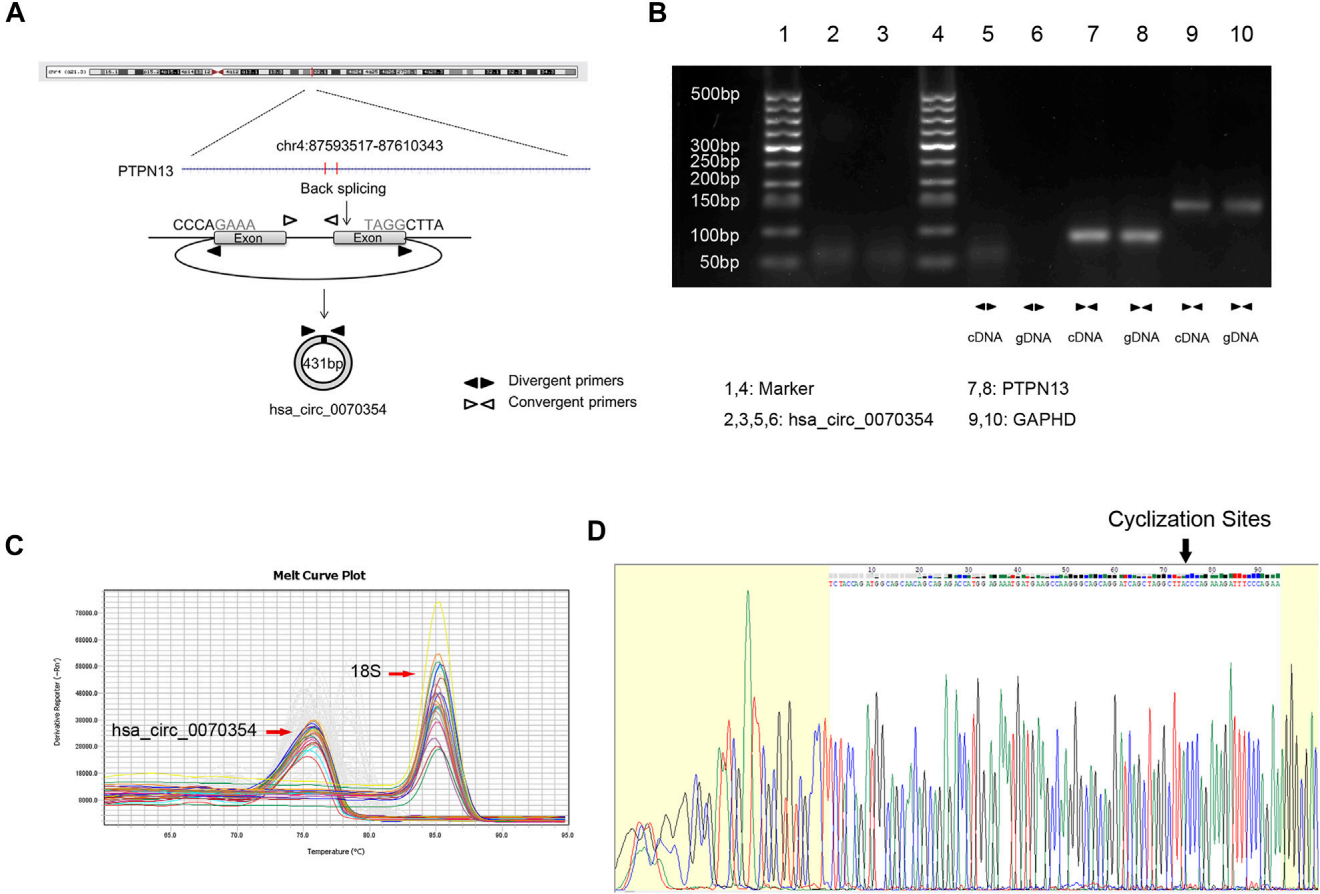
Identification of the molecular properties of hsa_circ_0070354. **(A)** Genomic location and splicing mode of hsa_circ_0070354. **(B)** Agarose gel electrophoresis was performed to detect the existence of hsa_circ_0070354 and PTPN13 from cDNA and gDNA in A549 cells using the divergent and convergent primers, respectively. Glyceraldehyde 3-phosphate dehydrogenase (GAPDH) as the negative control. **(C)** The PCR melting curve of hsa_circ_0070354. **(D)** Identification of hsa_circ_0070354 qPCR amplification products and the back-splice junction site by Sanger sequencing.

The high expression of hsa_circ_0070354 has a clear relationship with cancer, providing the possibility of hsa_circ_0070354 as a tumor biomarker. Due to the lack of previous studies, to further clarify the properties of hsa_circ_0070354, we have completed the following explorations. Initially, the Ensembl genome database (GRCh37/hg19) was applied to verify hsa_circ_0070354 located on chr4_87593517_87610343_+, with a predicted length of 431 bp (Figure 2A). In order to ensure that hsa_circ_0070354 was not *trans*-splicing or genomic rearrangement, we designed divergent and convergent primers to amplify the circRNA 0070354 and its source gene PTPN13 linear mRNA, respectively. The results of PCR amplification pointed out hsa_circ_0070354 was amplified from cDNA, excluding the existence of genomic DNA (gDNA), while convergent primers amplified PTPN13 from both cDNA and gDNA (Figure 2B). Consistently, agarose gel electrophoresis presented a single electrophoretic band, which was about the same size as the primer amplification product about 70 bp (Figure 2B). In addition, the presence of hsa_circ_0070354 was proved by RT-PCR (Figure 2C), and the back splicing junction of hsa_circ_0070354 in PCR products was confirmed by Sanger sequencing (Figure 2D).

**FIGURE 3:**
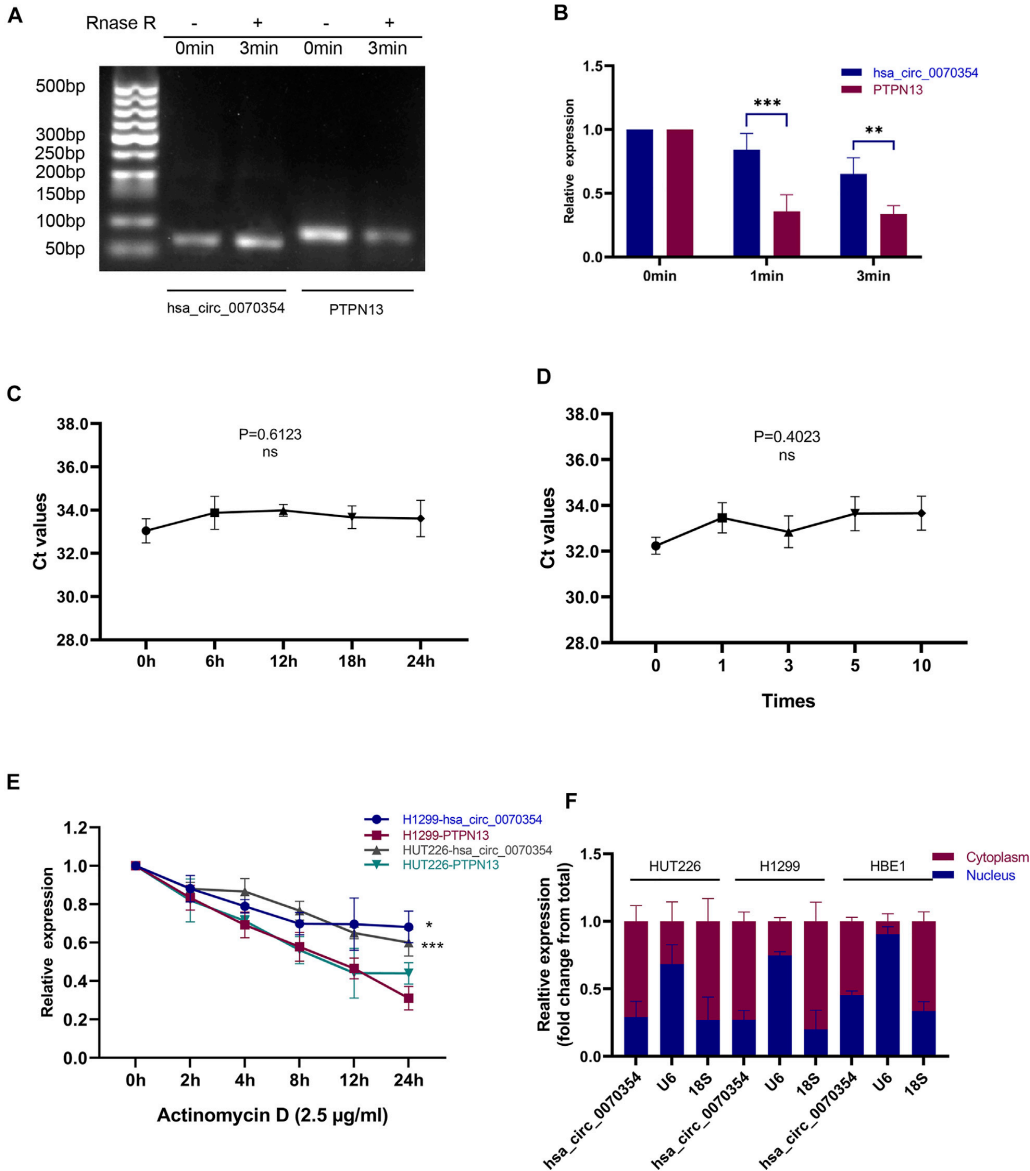
Characteristics of hsa_circ_0070354 as a serum tumor biomarker. **(A,B)** qRT-PCR analyses were conducted to determine the abundances of hsa_circ_0070354 and linear PTPN13 mRNA in A549 cells after treatment with RNase R at the indicated time points. **(C,D)** The expressions of hsa_circ_0070354 in mix serum which placed at room temperature at the indicated time points **(C)** or frozen and thawed 0, 1, 3, 5, 10 times **(D)**. **(E)** The RNA expressions of hsa_circ_0070354 and linear PTPN13 mRNA in A549 cells were analyzed by qRT-PCR after treatment with 2.5 μg/ml actinomycin D for 2, 4, 8, 12, 24 h. **(F)** Nuclear-cytoplasmic RNA fractionation assay showed that hsa_circ_0070354 was mainly localized in the cytoplasm of A549 cells.18S was used as a cytoplasmic protein control while U6 was considered as a nuclear control. *p < 0.05, **p < 0.01, ***p < 0.001.

Since circRNA is more stable than linear RNA and is not easily degraded by exonuclease, the expression of hsa_circ_0070354 and linear PTPN13 were detected by qRT-PCR after being treated with RNase R. The results presented that compared with linear PTPN13, the degradation of hsa_circ_0070354 was not significant (Figures 3A,B). The serum samples from 20 healthy controls were randomly mixed with equal volume, placed at room temperature for 24 h (Figure 3C), frozen, and thawed 0, 1, 3, 5, 10 times repeatedly (Figure 3D). Interestingly, the results showed no significant difference in cycle threshold (CT) value of hsa_circ_0070354 after these operations. As we are known, actinomycin D could inhibit the synthesis of new RNAs. To further evaluate the stability, the expression of hsa_circ_0070354 and linear PTPN13 was detected at the indicated time points after treatment with actinomycin D. The results signified that hsa_circ_0070354 had a longer half-life after treatment with actinomycin D (Figure 3E). All these results illustrated that hsa_circ_0070354 is a circRNA and the stability met the requirement of tumor biomarker. It is known that the circular RNA in the nucleus mainly regulates the transcription of parental genes, while the circular RNA in the cytoplasm primarily acts as a competitive endogenous RNA (ceRNA) (Rong et al., 2017; Xu et al., 2020). Through the nuclear-cytoplasmic separation experiment, we found that hsa_circ_0070354 is mainly located in the cytoplasm (Figure 3F) may be involved in post-transcriptional gene regulation, which is a new potential way for the treatment of NSCLC.

### Correlation Between High Expression of hsa_circ_0070354 and Clinicopathological Features

**FIGURE 4:**
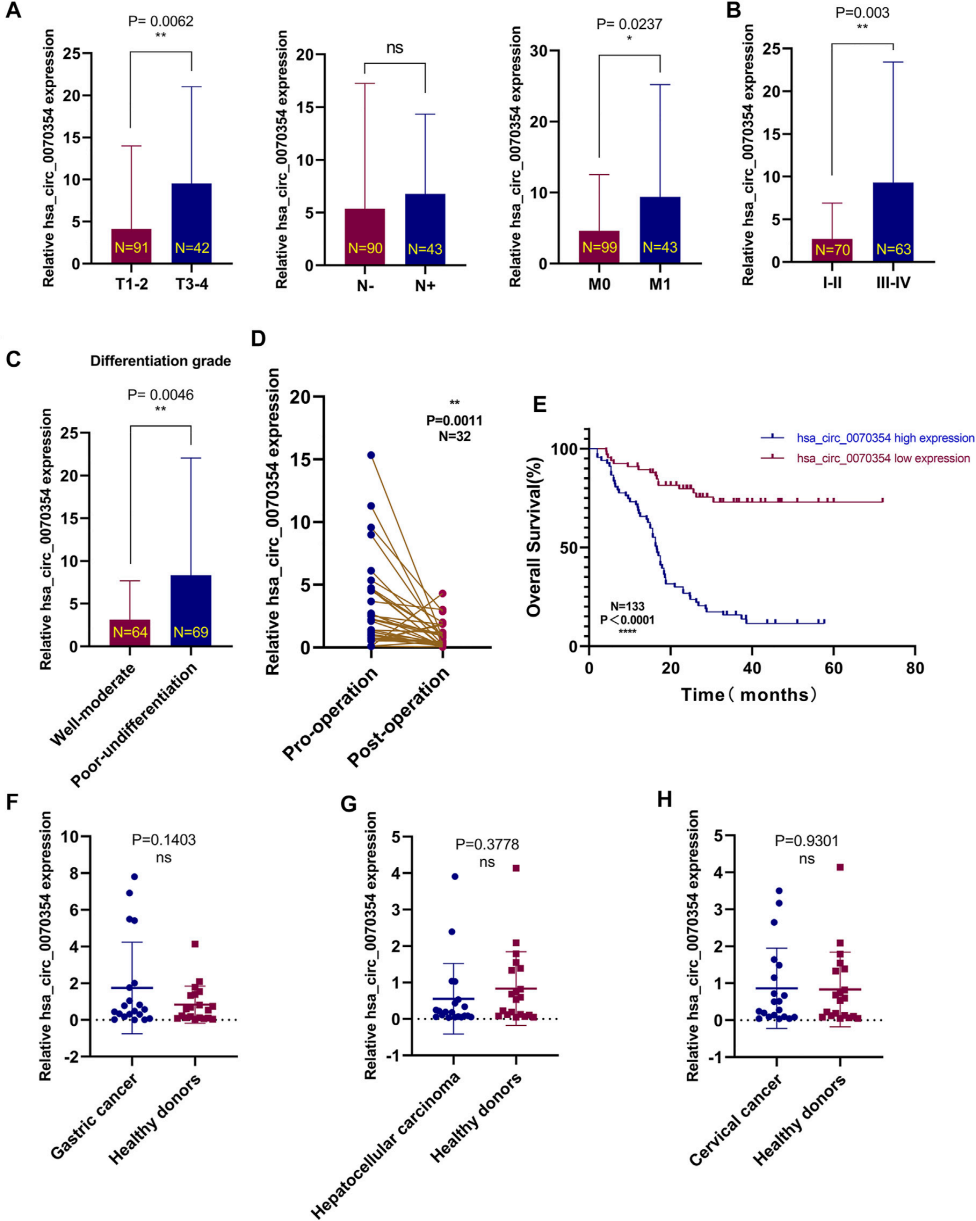
High hsa_circ_0070354 expression in the NSCLC serums and prognostic significance. **(A)** The expression levels of hsa_circ_0070354 in serum specimens of NSCLC at different stages of tumor size, lymph node metastasis, and distant metastasis. **(B,C)** The expression levels of hsa_circ_0070354 in serum specimens of NSCLC at different stages of TNM stage **(B)** and differentiation degree **(C)**. **(D)** The expression of hsa_circ_0070354 in patients with NSCLC before and after operation. **(E)** Prognostic analysis of hsa_circ_0070354 expression in 133 NSCLC patients. **(F–H)** The expression level of hsa_circ_0070354 in 20 cases of patients with gastric cancer, hepatocellular carcinoma, cervical cancer, and healthy donors, respectively. *p < 0.05, **p < 0.01, ***p < 0.001, ****p < 0.0001, NS *p* > 0.05.

For the purpose of analyzing the expression and clinical value of hsa_circ_0070354, we evaluated the relationship between the expression of hsa_circ_0070354 and clinicopathological features. The results illustrated that the expression of hsa_circ_0070354 was positively correlated with differentiation degree (*p* = 0.002), tumor size (*p* < 0.001), lymph node metastasis (*p* = 0.001), distant metastasis (*p* = 0.028), TNM stage (*p* < 0.001), Ki-67 expression (*p* = 0.005), the value of CEA (*p* = 0.038) and Cyfra21-1 (*p* = 0.007) by Chi-square test, but not with other clinicopathological parameters such as age, sex, smoking status, pathological type and so forth (Table 2). Secondly, the increase of hsa_circ_0070354 expression in serum of NSCLC patients was significantly different from that of TNM and differentiation in different stages (Figures 4A–C), which was basically consistent with the results in Table 2. Unfortunately, the difference between the actual expression of hsa_circ_0070354 and the N stage decreased statistically. When the tumor load decreased after the operation, the expression of hsa_circ_0070354 was conspicuous lower than that before the operation (Figure 4D). Univariate and multivariate Cox proportional hazards regression models were performed to assess the impacts of the clinical variables on the OS of NSCLC patients (Table 3). The multivariate Cox analysis results indicated that the high expression of hsa_circ_0070354 was an independent predictor of poor prognosis in patients with NSCLC (HR = 5.087, 95% CI: 2.442–10.600, *p* < 0.001). Importantly, the Kaplan-Meier survival curve showed that the patients with hsa_circ_0070354 high expression had a significantly worse prognosis than those with hsa_circ_0070354 low (*p* < 0.001, log-rank test; Figure 4E). Through the qRT-PCR, we found that there was no statistically significant difference between the patients with gastric cancer (*N* = 20, *p* = 0.1403), hepatocellular carcinoma (*N* = 20, *p* = 0.3788), cervical cancer (*N* = 20, *p* = 0.9301), and healthy donors (Figures 4F–H). Given its overexpression in NSCLC, it would be appropriate to suggest that hsa_circ_0070354 was NSCLC-specific through serum screening of other small tumor samples. In conclusion, hsa_circ_0070354 likely participates in the progression of NSCLC, and the high expression of hsa_circ_0070354 can be used as a poor prognostic factor.

**TABLE 2:** The association between hsa_circ_0070354 expression and clinicopathologic parameters in 133 NSCLC specimens.

Parameters	Total	hsa_circ_0070354 expression		*p* Value
		Low (<median)	High (≥median)	
Age				0.850
< 55	39	20	19	
≥ 55	94	46	48	
Gender				1.000
Male	63	31	32	
Female	70	35	35	
Smoking status				0.581
Non-smoker	89	46	43	
Smoker	44	20	24	
Pathological Type				0.179
Suqamous	38	15	23	
Adenocarcinoma	95	51	44	
Differentiation grade				0.002**
Well-moderate	64	41	23	
Poor-undifferentiation	69	25	44	
Tumor size				0.000***
<5 cm	91	57	34	
≥5 cm	42	9	33	
Lymph node metastasis				0.001**
Negative	90	54	36	
Positive	43	12	31	
Distant metastasis				0.028*
Negative	99	55	44	
Positive	34	11	23	
TNM stage				0.000***
I–II	70	54	16	
III–IV	63	12	51	
Ki-67 expression				0.005**
< 30%	78	47	31	
≥ 30%	55	19	36	
Pleural invasion				0.186
Negative	93	50	43	
Positive	40	16	24	
Nerve invasion				0.244
Negative	131	64	67	
Positive	2	2	0	
Vascular invasion				0.682
Negative	102	52	50	
Positive	31	14	17	
Spread through Air Spaces				0.858
Negative	83	42	41	
Positive	50	24	26	
CEA				0.038*
Negative(<5 ng/ml)	66	39	27	
Positive(≥5 ng/ml)	67	27	40	
SCC				0.469
Negative(<1.5 μg/L)	86	45	41	
Positive(≥1.5 μg/L)	47	21	26	
Cyfra21-1				0.007**
Negative(<2.08 ng/ml)	94	54	40	
Positive(≥2.08 ng/ml)	39	12	27	
CA199				0.115
Negative(<37 U/ml)	126	65	61	
Positive(≥37 U/ml)	7	1	6	
Serum ferritsn				0.483
Negative(<204 μg/L)	78	41	37	
Positive(≥204 μg/L)	55	25	30	

Statistical analyses were performed by the Pearson χ^2^ test. *p < 0.05, **p < 0.01, ***p < 0.001 was considered significant.

**TABLE 3:** Univariate and multivariate Cox regression analysis of has_circ_0070354 and clinical variables predicting survival from 133 NSCLC specimens.

Parameter	Univariate analysis			Multivariate analysis		
	HR	95% CI	*p* Value	HR	95% CI	*p* Value
Age (<55 vs ≥55)	1.252	0.742–2.113	0.401	—	—	—
Gender (Male vs. Female)	0.800	0.505–1.268	0.343	—	—	—
Smoking status (Non-smoker vs. Smoker)	0.524	0.304–0.903	0.020*	1.571	0.720–3.426	0.256
Pathological Type (Suqamous vs. Adenocarcinoma)	1.423	0.835–2.423	0.194	-	—	—
Differentiation grade (Well-moderate vs. Poor-undifferentiation)	2.445	1.498–3.990	<0.001***	1.054	0.477–2.328	0.896
Tumor size (<5 cm vs. ≥5 cm)	2.526	1.592–4.007	<0.001***	1.336	0.717–2.487	0.361
Lymph node metastasis (Negative vs. Positive)	2.935	1.846–4.666	<0.001***	0.664	0.339–1.303	0.234
Distant metastasis (Negative vs. Positive)	2.611	1.622–4.201	<0.001***	1.575	0.578–4.287	0.347
TNM stage (I–II vs. III–IV)	5.666	3.309–9.700	<0.001***	0.317	0.112–0.892	0.029*
Ki-67 expression (<30% vs. ≥30%)	2.459	1.541–3.924	<0.001***	0.977	0.453–2.108	0.953
Pleural invasion (Negative vs. Positive)	1.335	0.827–2.156	0.237	—	—	—
Nerve invasion (Negative vs. Positive)	0.854	0.119–6.156	0.876	—	—	—
Vascular invasion (Negative vs. Positive)	2.526	1.549–4.118	<0.001***	0.462	0.140–1.519	0.203
Spread through Air Spaces (Negative vs. Positive)	1.752	1.103–2.781	0.017*	0.793	0.324–1.940	0.611
CEA (<5 ng/ml vs. ≥5 ng/ml)	2.147	1.328–3.473	0.002**	1.260	0.641–2.478	0.502
SCC (<1.5 μg/L vs. ≥1.5 μg/L)	1.070	0.665–1.721	0.781	—	—	—
Cyfra21-1 (<2.08 ng/ml vs. ≥2.08 ng/ml)	1.668	1.031–2.697	0.037*	1.103	0.592–2.057	0.757
CA199 (<37 U/ml vs. ≥37 U/ml)	2.435	1.050–5.646	0.038*	0.715	0.235–2.169	0.553
Serum ferritsn (<204 μg/L vs. ≥204 μg/L)	1.381	0.872–2.186	0.169	—	—	—
has_circ_0070354 (Negative vs. Positive)	5.641	3.222–9.876	<0.001***	5.087	2.442–10.600	<0.001***

*p < 0.05, **p < 0.01, ***p < 0.001 was considered significant.

### Evaluation of the Diagnostic Accuracy of Serum hsa_circ_0070354 Independent and Combined Diagnostic Model in NSCLC

**FIGURE 5:**
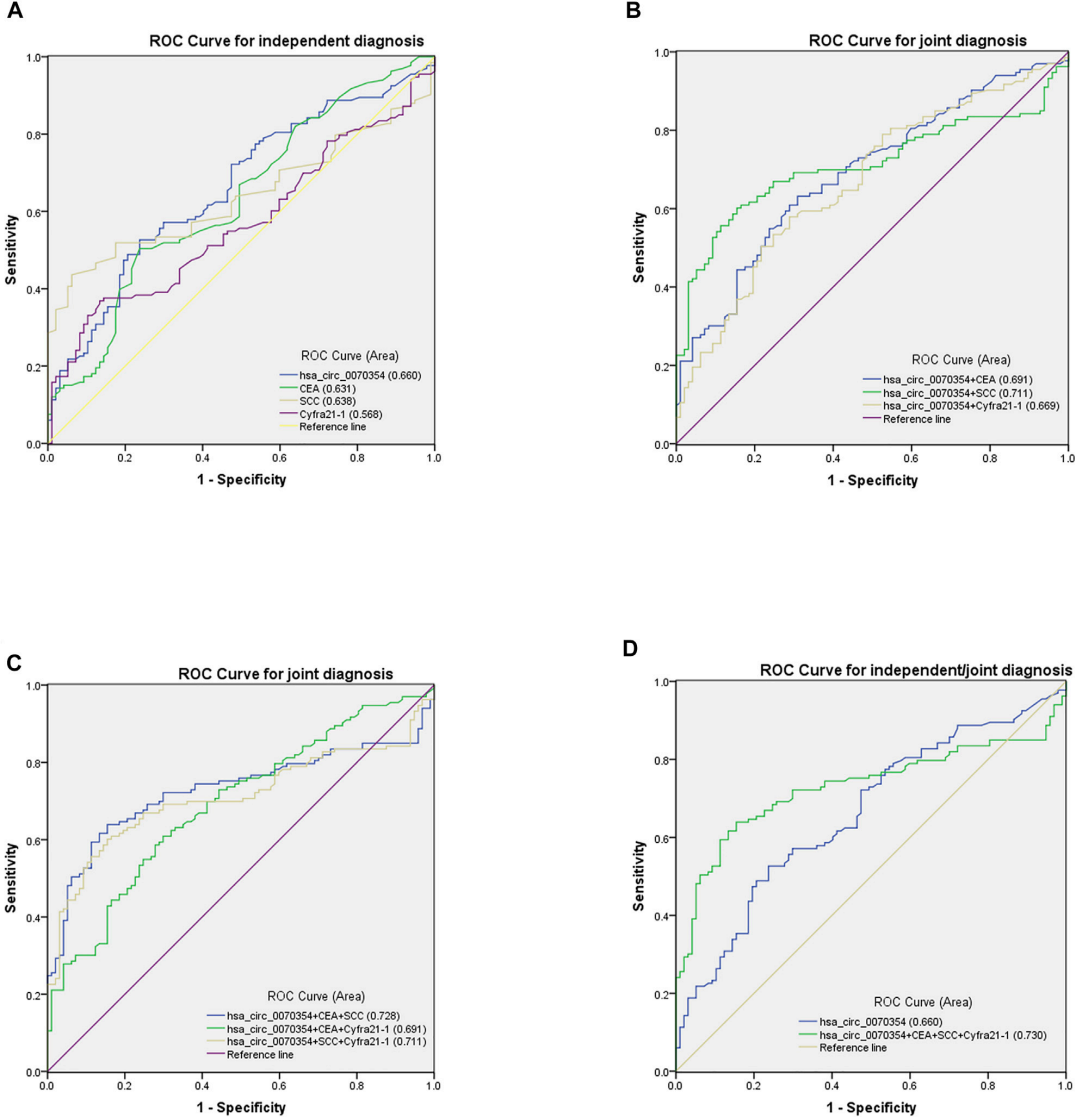
ROC curve evaluated the diagnostic value of hsa_circ_0070354 in NSCLC. **(A)** ROC curves for diagnosis using serum hsa_circ_0070354, CEA, SCC, and Cyfra21-1 between NSCLC patients (*N* = 133) and healthy controls (*N* = 97) independently. **(B–D)** ROC curves for combined diagnostic efficacy of serum hsa_circ_0070354, CEA, SCC, and Cyfra21-1 in distinguishing NSCLC patients with healthy donors.

In previous essays, hsa_circ_0070354 has been confirmed to have the molecular properties of tumor biomarkers and was related to poor tumor prognosis. And yet, its efficacy in the diagnosis of NSCLC needs to be further explored. Compared with the mature lung cancer markers such as CEA and SCC, the diagnostic efficiency of hsa_circ_0070354 is the key factor in evaluating it as a novel diagnostic and predictive biomarker. The ROC curve showed that the AUC of hsa_circ_0070354 was 0.660 (95% CI: 0.589–0.730), which was higher than 0.631 of CEA (95% CI: 0.559–0.703), 0.638 of SCC (95% CI: 0.557–0.709), and 0.568 of Cyfra21-1 (95% CI: 0.495–0.642) (Figure 5A). Figures 5B,C demonstrated the AUC of hsa_circ_0070354 combined with the other three tumor markers, respectively. Compared with the single diagnosis, the AUC of the combined diagnosis of hsa_circ_0070354 and CEA increased to 0.691 (95% CI: 0.623–0.758), as well as the AUC of combined diagnosis with SCC and Cyfra21-1 increased to 0.711 (95% CI: 0.644–0.779) and 0.669 (95% CI: 0.599–0.733) respectively, which were both higher than the single diagnosis (Figure 5B). Similarly, the three-combination diagnosis of AUC was remarkably higher than that of a single diagnosis (Figure 5C). What is particularly striking is that in Figure 5D, the AUC of the combination of hsa_circ_0070354 and the other three mature tumor markers reached 0.730 (95% CI: 0.663–0.796), which was significantly higher than that of the single diagnosis of hsa_circ_0070354, and also higher than the combined diagnosis of the two or three. Subsequently, we also analyzed the sensitivity (SEN), specificity (SPE), overall accuracy (ACCU), positive predictive value (PPV), as well as negative predictive value (NPV) of single and combined diagnostic models (Table 4). According to our statistics, the SEN, SPE, ACCU, PPV and NPV of hsa_circ_0070354 were 52.63, 76.29, 62.61, 75.27 and 54.01%, respectively, while the combined diagnosis of the four tumor markers were 63.91, 84.54, 72.61, 85.00 and 63.08%. Therefore, hsa_circ_0070354 is superior to traditional tumor markers in diagnosis, and the combined diagnosis has higher sensitivity and specificity for the diagnosis of NSCLC.

**TABLE 4:** Use the expression levels of has_circ_0070354, CEA, SCC and Cyfra21-1 to distinguish NSCLC patients from healthy donors.

	SEN (%)	SPE (%)	ACCU (%)	PPV (%)	NPV (%)
has_circ_0070354	52.63 (70/133)	76.29 (74/97)	62.61 (144/230)	75.27 (70/93)	54.01 (74/137)
CEA	50.38 (67/133)	77.32 (75/97)	61.74 (142/230)	75.28 (67/89)	53.19 (75/141)
SCC	35.34 (47/133)	94.85 (92/97)	60.43 (139/230)	90.38 (47/52)	51.69 (92/178)
Cyfra21-1	29.32 (39/133)	90.72 (88/97)	55.22 (127/230)	81.25 (39/48)	48.35 (88/182)
has_circ_0070354 + CEA	63.16 (84/133)	69.07 (67/97)	65.65 (151/230)	73.68 (84/114)	57.76 (67/116)
has_circ_0070354 + SCC	60.15 (80/133)	74.23 (72/97)	66.09 (152/230)	84.21 (80/95)	57.60 (72/125)
has_circ_0070354 + Cyfra21-1	57.89 (77/133)	71.13 (69/97)	63.48 (146/230)	73.33 (77/105)	55.20 (69/125)
has_circ_0070354 + CEA + SCC	63.91 (85/133)	84.54 (82/97)	72.61 (167/230)	85.00 (85/100)	63.08 (82/130)
has_circ_0070354 + CEA + Cyfra21-1	60.90 (81/133)	70.10 (68/97)	64.78 (149/230)	73.64 (81/110)	56.67 (68/120)
has_circ_0070354 + SCC + Cyfra21-1	60.15 (80/133)	84.54 (82/97)	70.43 (162/230)	84.21 (80/95)	60.74 (82/135)
has_circ_0070354 + CEA + SCC + Cyfra21-1	63.91 (85/133)	84.54 (82/97)	72.61 (167/230)	85.00 (85/100)	63.08 (82/130)

SEN, sensitivity; SPE, specificity; ACCU, overall accuracy; PPV, positive predictive value; NPV, negative predictive value.

### Exploration of the Downstream and Function Prediction of hsa_circ_0070354

**FIGURE 6:**
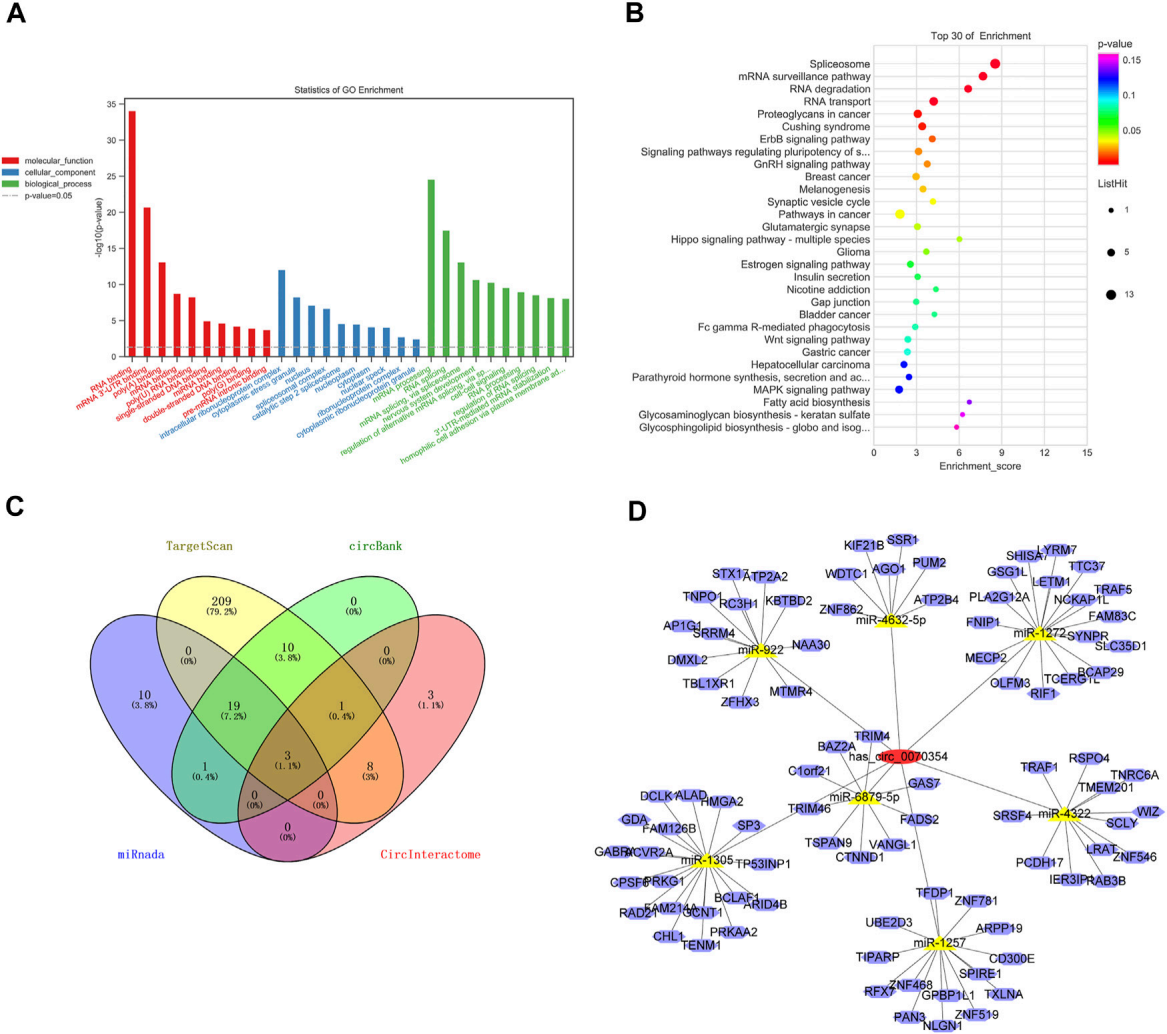
Exploration of the downstream regulatory network of hsa_circ_0070354 in NSCLC. **(A)** GO analysis of the hsa_circ_0070354 target genes enriched in molecular function (red), cellular component (blue), and biological process (green). **(B)** Bubble chart of KEGG analysis of candidate hsa_circ_0070354 different target genes expressed of enriched pathways. **(C)** Venny diagram evaluated the overlapped genes between TargetScan, circBank, miRandan, and CircInteractome predictions. **(D)** Prediction of the circRNA-miRNA-mRNA network map of hsa_circ_0070354 by Cytoscape analysis. The red oval represents hsa_circ_0070354, and the yellow triangle represents seven miRNAs that could interact with hsa_circ_0070354, while the purple oval represents the target mRNA of the corresponding miRNA.

Previous studies have implied that circRNAs have four main functions: acting as a miRNA sponge, interacting with RNA binding proteins, regulating gene transcription, and encoding peptides. circRNAs contain a massive number of miRNA binding sites, which act as miRNA sponges and adjust gene expression indirectly (Huang et al., 2021a). Hsa_circ_0070354 was mainly located in the cytoplasm and may participate in the competitive inhibition of endogenous RNA as a microRNA sponge. Yet, its participation in the occurrence and development of NSCLC is a very complex process, which is worth our further in-depth study. Here, we analyze its potential function and regulation mechanism through the prediction of the relevant database. The Gene Ontology (GO) functional enrichment analysis of the target gene shows that 354 may play a potential role in RNA binding, splicing, and processing (Figure 6A). The enrichment analysis of the Kyoto Encyclopedia of Genes and Genomes (KEGG) signal pathway showed that hsa_circ_0070354 was significantly enriched in a variety of tumors, and the ErbB and MAPK signal pathway was also the focus of our subsequent attention (Figure 6B). The Venny diagram shows our prediction of downstream miRNAs in four common databases. The three miRNAs obtained by repeated intersection are miR-1257, miR-1272, and miR-1305 (Figure 6C). According to the results of databases and bioinformatics analysis, we predict the possible circRNA-miRNA-mRNA axis. As shown in Figures 5A,D total of 7 miRNAs (3 above, also including miR-922, miR-4322, miR-4632-5p, and miR-6879-5p) and their corresponding mRNAs are presented, which may provide a research direction for the progress of NSCLC regulation by hsa_circ_0070354. In any case, based on the above results, more experimental work is needed to confirm the regulation mechanism of hsa_circ_0070354.

## Discussion

Generally, the incidence and malignancy of NSCLC are high, and the 5-year survival rate is lower than other malignant tumors, which is mainly due to the enormous heterogeneity of NSCLC and the insufficient understanding of the biological of NSCLC, leading to the lack of effective biomarkers for diagnosis and targeted therapies (Fu et al., 2020). Besides, the survival of NSCLC is closely related to staging. Studies have shown that as patients develop from stage I to IV, the 5-year survival rate of NSCLC decreases from 82 to 6% (Goldstraw et al., 2016). Most of the patients have been in the advanced or locally advanced stage at the time of diagnosis and have missed the best opportunity for surgical treatment, mainly conservative palliative treatment. Despite that, the research on targeted therapy and immunotherapy for advanced patients is advancing by leaps and bounds. The lack of targets and low mutation rate is still the treatment dilemma. The high cost of immunotherapy and unpredictable side effects also deter many patients. Even so, the increased drug resistance of targeted therapy and immunotherapy is also the limitation of treatment, and tumors develop irreversible outcomes (Hong et al., 2020). For these reasons, early detection and improving the accuracy of diagnosis are conducive to improve the prognosis and survival rate of patients, which not only depends on the popularization of physical examination and computerized tomography (CT) but also requires the exploration and research of humoral tumor biomarkers which are non-radiation, dynamic monitoring, simple and easy to popularize. It is an urgent issue to identify new specific biomarkers for NSCLC to aid in diagnosis and clinical decision.

CircRNA was first observed in the 1970s (Hsu and Coca-Prados, 1979), but its biological function was considered unclear for a long time (Cocquerelle et al., 1993; Zaphiropoulos, 1997). Until 2012, with the advancement of high-throughput sequencing and bioinformatics analysis, the distribution and function of circRNAs in different cells and tissues were progressively discovered, not just an idle by-product of mRNA splicing (Djebali et al., 2012; Salzman et al., 2012). Since then, the researchers focused on circRNAs have shifted attention to clarifying their associations with human diseases, including cancers. Some kinds of literature have reported that the abnormal expressions of circRNAs were associated with the clinical characteristics of tumors and might become biomarkers of NSCLC (Kristensen et al., 2018; Qu et al., 2018; Di et al., 2019). In this study, we determined for the first time that hsa_circ_0070354 was higher in tumor tissues, cells, and serum than in adjacent normal tissues, cell lines, or healthy human sera. At the same time, we found that the high expression of hsa_circ_0070354 was significantly positively correlated with the late TNM stage and poor differentiation of NSCLC, indicating a poor prognosis and survival time. Combined with the biological characteristics of sensitivity, stability, and ease to be detected in body fluids, hsa_circ_0070354 would be a promising biomarker of NSCLC and has a bright prospect for early diagnosis and treatment of lung cancer in the future. The relevant information at the beginning of our research came from some circRNA databases, such as circBank, circBase and circular RNA Interactome. Through circBase, we found that hsa_circ_0070354 studies included Rybak2015 and Salzman2013. Except for A549 cell line, which was consistent with our experimental results, it was only expressed in the brain parenchyma, such as the cerebellum, occipital_lobe and frontal_cortex, but no evidence was found in other cancers. In the exosomes database, such as ExoRbase, we have not queried the relevant included information. The existing information supports the specific expression of hsa_circ_0070354 in NSCLCs. In addition, the transition from a single biomarker to the joint diagnosis of multiple biomarkers is the development direction of tumor markers. Our results revealed that the combined diagnosis of multiple tumor markers has significantly higher sensitivity and specificity than a single biomarker. The combination of hsa_circ_0070354 with other tumor markers is expected to improve further the accuracy of hsa_circ_0070354 in the diagnosis, prognosis, and prediction of NSCLC. Generally, circRNAs are formed by reverse splicing of pre-mRNAs through several acknowledged mechanisms, namely lariat-driven circularization (Eger et al., 2018), intron pairing-driven circularization (Liu et al., 2019b), RBP-mediated circularization (Conn et al., 2015), and intronic circularization (Zhang et al., 2013). The relationship between host gene and circRNA is complex and diverse. However, the expression levels of circRNAs and the abundance of mRNA produced by the parental gene were not significantly related (Barrett and Salzman, 2016; Chen et al., 2019). By querying TCGA resource, it can be easily noted that the host gene of hsa_circ_0070354 (PTPN13) show negative association with cancer prognosis (HR<1), which is contrary to hsa_circ_0070354 (HR>1). Due to the limitations of database speculation and the incidence characteristics of NSCLC in Asia, the expression of PTPN13 in Chinese population needs further large sample study. This different expression trend inspired us to explore the further regulatory axis between hsa_circ_0070354 and its host gene PTPN13 in depth.

In the study of the mechanisms of circRNAs, the ceRNA hypothesis proposes that RNA transcripts share the same miRNA response elements, leading to the competition with miRNA combinations and then regulating the expressions (Salmena et al., 2011). In our study, we combined the results of bioinformatics analysis and sequencing to screen the miRNA and mRNA binding to hsa_circ_0070354. Unfortunately, the high expression of hsa_circ_0070354 in the biological, behavioral regulation of NSCLC remains unclear. The analysis between hsa_circ_0070354 and clinicopathological features suggested it was related to tumor size and metastasis, corresponding to biological behaviors such as cell proliferation and invasion, which provides a direction for further exploration. Whether the hsa_circ_0070354-miRNA-mRNA axis is involved in regulating NSCLC cell proliferation, migration and invasion will be the direction of our next exploration and study. Although no study has confirmed that circRNA would be a new therapeutic target for tumors, the effects of circRNAs on the proliferation, metastasis, and drug resistance of lung cancer indicate that they may become a target of therapeutic, which is worthy of our profound understanding. CircRNAs are expected to make significant contributions to cancer prevention, diagnosis, and treatment in the future.

## Data Availability

The data presented in the study are deposited in the NCBI SRA repository, accession number PRJNA 781220.

## Abbreviations

ACCU, overall accuracy; AUC, area under the curve; cDNA, complementary DNA; ceRNA, competitive endogenous RNA; circRNAs, circular RNAs; COPD, chronic obstructive pulmonary disease; GO, Gene Ontology; gDNA, genomic DNA; KEGG, Kyoto Encyclopedia of Genes and Genomes; NPV, negative predictive value; NSCLC, non-small cell lung cancer; OS, overall survival; PD-1, programmed cell death protein-1; PPV, positive predictive value; ROC, receiver operating characteristic; SEN, sensitivity; SPE, specificity.

## References

[B1] Barrett S. P. Salzman J. (2016). Circular RNAs: Analysis, Expression and Potential Functions. Development 143 (11), 1838–1847. 10.1242/dev.128074 27246710 PMC4920157

[B2] Chaft J. E. Rimner A. Weder W. Azzoli C. G. Kris M. G. Cascone T. (2021). Evolution of Systemic Therapy for Stages I-III Non-metastatic Non-small-cell Lung Cancer. Nat. Rev. Clin. Oncol. 18 (9), 547–557. 10.1038/s41571-021-00501-4 33911215 PMC9447511

[B3] Chen S. Huang V. Xu X. Livingstone J. Soares F. Jeon J. (2019). Widespread and Functional RNA Circularization in Localized Prostate Cancer. Cell 176 (4), 831–843. 10.1016/j.cell.2019.01.025 30735634

[B4] Cocquerelle C. Mascrez B. Hétuin D. Bailleul B. (1993). Mis-splicing Yields Circular RNA Molecules. FASEB J. 7 (1), 155–160. 10.1096/fasebj.7.1.7678559 7678559

[B5] Conn S. J. Pillman K. A. Toubia J. Conn V. M. Salmanidis M. Phillips C. A. (2015). The RNA Binding Protein Quaking Regulates Formation of circRNAs. Cell 160 (6), 1125–1134. 10.1016/j.cell.2015.02.014 25768908

[B6] Di X. Jin X. Li R. Zhao M. Wang K. (2019). CircRNAs and Lung Cancer: Biomarkers and Master Regulators. Life Sci. 220, 177–185. 10.1016/j.lfs.2019.01.055 30711537

[B7] Djebali S. Davis C. A. Merkel A. Dobin A. Lassmann T. Mortazavi A. (2012). Landscape of Transcription in Human Cells. Nature 489 (7414), 101–108. 10.1038/nature11233 22955620 PMC3684276

[B8] Eger N. Schoppe L. Schuster S. Laufs U. Boeckel J.-N. (2018). Circular RNA Splicing. Adv. Exp. Med. Biol. 1087, 41–52. 10.1007/978-981-13-1426-1_4 30259356

[B9] ENCODE Project Consortium (2012). An Integrated Encyclopedia of DNA Elements in the Human Genome. Nature 489 (7414), 57–74. 10.1038/nature11247 22955616 PMC3439153

[B10] Ferlay J. Shin H.-R. Bray F. Forman D. Mathers C. Parkin D. M. (2010). Estimates of Worldwide burden of Cancer in 2008: GLOBOCAN 2008. Int. J. Cancer 127 (12), 2893–2917. 10.1002/ijc.25516 21351269

[B11] Fu Y. Huang L. Tang H. Huang R. (2020). Hsa_circRNA_012515 Is Highly Expressed in NSCLC Patients and Affects its Prognosis. Cancer Manag. Res. 12, 1877–1886. 10.2147/CMAR.S245525 32210630 PMC7075336

[B12] Glažar P. Papavasileiou P. Rajewsky N. (2014). circBase: a Database for Circular RNAs. RNA 20 (11), 1666–1670. 10.1261/rna.043687.113 25234927 PMC4201819

[B13] Goldstraw P. Chansky K. Crowley J. Rami-Porta R. Asamura H. Eberhardt W. E. (2016). The IASLC Lung Cancer Staging Project: Proposals for Revision of the TNM Stage Groupings in the Forthcoming (Eighth) Edition of the TNM Classification for Lung Cancer. J. Thorac. Oncol. 11 (1), 39–51. 10.1016/j.jtho.2015.09.009 26762738

[B14] Hang D. Zhou J. Qin N. Zhou W. Ma H. Jin G. (2018). A Novel Plasma Circular RNA circFARSA Is a Potential Biomarker for Non-small Cell Lung Cancer. Cancer Med. 7 (6), 2783–2791. 10.1002/cam4.1514 29722168 PMC6010816

[B15] Hansen T. B. Jensen T. I. Clausen B. H. Bramsen J. B. Finsen B. Damgaard C. K. (2013). Natural RNA Circles Function as Efficient microRNA Sponges. Nature 495 (7441), 384–388. 10.1038/nature11993 23446346

[B16] Hong W. Xue M. Jiang J. Zhang Y. Gao X. (2020). Circular RNA Circ-CPA4/Let-7 miRNA/PD-L1 axis Regulates Cell Growth, Stemness, Drug Resistance and Immune Evasion in Non-small Cell Lung Cancer (NSCLC). J. Exp. Clin. Cancer Res. 39 (1), 149. 10.1186/s13046-020-01648-1 32746878 PMC7397626

[B17] Hsu M.-T. Coca-Prados M. (1979). Electron Microscopic Evidence for the Circular Form of RNA in the Cytoplasm of Eukaryotic Cells. Nature 280 (5720), 339–340. 10.1038/280339a0 460409

[B18] Huang L. Rong Y. Tang X. Yi K. Wu J. Wang F. (2021). Circular RNAs Are Promising Biomarkers in Liquid Biopsy for the Diagnosis of Non-small Cell Lung Cancer. Front. Mol. Biosci. 8, 625722. 10.3389/fmolb.2021.625722 34136531 PMC8201604

[B19] Huang Y. Zhang H. Gu X. Qin S. Zheng M. Shi X. (2021). Elucidating the Role of Serum tRF-31-U5YKFN8DYDZDD as a Novel Diagnostic Biomarker in Gastric Cancer (GC). Front. Oncol. 11, 723753. 10.3389/fonc.2021.723753 34497770 PMC8419412

[B20] Jeck W. R. Sharpless N. E. (2014). Detecting and Characterizing Circular RNAs. Nat. Biotechnol. 32 (5), 453–461. 10.1038/nbt.2890 24811520 PMC4121655

[B21] Jeck W. R. Sorrentino J. A. Wang K. Slevin M. K. Burd C. E. Liu J. (2013). Circular RNAs Are Abundant, Conserved, and Associated with ALU Repeats. RNA 19 (2), 141–157. 10.1261/rna.035667.112 23249747 PMC3543092

[B22] Kamel L. M. Atef D. M. Mackawy A. M. H. Shalaby S. M. Abdelraheim N. (2019). Circulating Long Non‐coding RNA GAS5 and SOX2OT as Potential Biomarkers for Diagnosis and Prognosis of Non‐small Cell Lung Cancer. Biotechnol. Appl. Biochem. 66 (4), 634–642. 10.1002/bab.1764 31077615

[B23] Kristensen L. S. Hansen T. B. Venø M. T. Kjems J. (2018). Circular RNAs in Cancer: Opportunities and Challenges in the Field. Oncogene 37 (5), 555–565. 10.1038/onc.2017.361 28991235 PMC5799710

[B24] Liu Y.-T. Han X.-H. Xing P.-Y. Hu X.-S. Hao X.-Z. Wang Y. (2019). Circular RNA Profiling Identified as a Biomarker for Predicting the Efficacy of Gefitinib Therapy for Non-small Cell Lung Cancer. J. Thorac. Dis. 11 (5), 1779–1787. 10.21037/jtd.2019.05.22 31285870 PMC6588778

[B25] Liu K. S. Pan F. Mao X. D. Liu C. Chen Y. J. (2019). Biological Functions of Circular RNAs and Their Roles in Occurrence of Reproduction and Gynecological Diseases. Am. J. Transl. Res. 11 (1), 1–15. 30787966 PMC6357300

[B26] Liu Z. H. Yang S. Z. Chen X. T. Shao M. R. Dong S. Y. Zhou S. Y. (2020). Correlations of Hsa_circ_0046264 Expression with Onset, Pathological Stage and Chemotherapy Resistance of Lung Cancer. Eur. Rev. Med. Pharmacol. Sci. 24 (18), 9511–9521. 10.26355/eurrev_202009_23036 33015793

[B27] Lu H. Xie X. Chen Q. Cai S. Liu S. Bao C. (2021). Clinical Significance of circPVT1 in Patients with Non-small Cell Lung Cancer Who Received Cisplatin Combined with Gemcitabine Chemotherapy. Tumori 107 (3), 030089162094194. 10.1177/0300891620941940 32734834

[B28] Ma L.-R. Li J.-X. Tang L. Li R.-Z. Yang J.-S. Sun A. (2021). Immune Checkpoints and Immunotherapy in Non-Small Cell Lung Cancer: Novel Study Progression, Challenges and Solutions (Review). Oncol. Lett. 22 (5), 787. 10.3892/ol.2021.13048 34594428 PMC8456509

[B29] Memczak S. Jens M. Elefsinioti A. Torti F. Krueger J. Rybak A. (2013). Circular RNAs Are a Large Class of Animal RNAs with Regulatory Potency. Nature 495 (7441), 333–338. 10.1038/nature11928 23446348

[B30] Nigro J. M. Cho K. R. Fearon E. R. Kern S. E. Ruppert J. M. Oliner J. D. (1991). Scrambled Exons. Cell 64 (3), 607–613. 10.1016/0092-8674(91)90244-s 1991322

[B31] Pei X. Chen S.-W. Long X. Zhu S.-Q. Qiu B.-Q. Lin K. (2020). circMET Promotes NSCLC Cell Proliferation, Metastasis, and Immune Evasion by Regulating the miR-145-5p/CXCL3 axis. Aging 12 (13), 13038–13058. 10.18632/aging.103392 32614785 PMC7377868

[B32] Peng L. Yuan X. Q. Li G. C. (2015). The Emerging Landscape of Circular RNA ciRS-7 in Cancer (Review). Oncol. Rep. 33 (6), 2669–2674. 10.3892/or.2015.3904 25873049

[B33] Qian H. Zhang Y. Xu J. He J. Gao W. (2021). Progress and Application of Circulating Tumor Cells in Non-small Cell Lung Cancer. Mol. Ther. Oncol. 22, 72–84. 10.1016/j.omto.2021.05.005 PMC8408556 34514090

[B34] Qu S. Liu Z. Yang X. Zhou J. Yu H. Zhang R. (2018). The Emerging Functions and Roles of Circular RNAs in Cancer. Cancer Lett. 414, 301–309. 10.1016/j.canlet.2017.11.022 29174799

[B35] Rong D. Sun H. Li Z. Liu S. Dong C. Fu K. (2017). An Emerging Function of circRNA-miRNAs-mRNA axis in Human Diseases. Oncotarget 8 (42), 73271–73281. 10.18632/oncotarget.19154 29069868 PMC5641211

[B36] Rybak-Wolf A. Stottmeister C. Glažar P. Jens M. Pino N. Giusti S. (2015). Circular RNAs in the Mammalian Brain Are Highly Abundant, Conserved, and Dynamically Expressed. Mol. Cel 58 (5), 870–885. 10.1016/j.molcel.2015.03.027 25921068

[B37] Salmena L. Poliseno L. Tay Y. Kats L. Pandolfi P. P. (2011). A ceRNA Hypothesis: the Rosetta Stone of a Hidden RNA Language? Cell 146 (3), 353–358. 10.1016/j.cell.2011.07.014 21802130 PMC3235919

[B38] Salzman J. Gawad C. Wang P. L. Lacayo N. Brown P. O. (2012). Circular RNAs Are the Predominant Transcript Isoform from Hundreds of Human Genes in Diverse Cell Types. PLoS One 7 (2), e30733. 10.1371/journal.pone.0030733 22319583 PMC3270023

[B39] Wan L. Zhang L. Fan K. Cheng Z.-X. Sun Q.-C. Wang J.-J. (2016). Circular RNA-ITCH Suppresses Lung Cancer Proliferation via Inhibiting the Wnt/β-Catenin Pathway. Biomed. Res. Int. 2016, 1–11. 10.1155/2016/1579490 PMC5013215 27642589

[B40] Wang L. Tong X. Zhou Z. Wang S. Lei Z. Zhang T. (2018). Circular RNA Hsa_circ_0008305 (circPTK2) Inhibits TGF-β-Induced Epithelial-Mesenchymal Transition and Metastasis by Controlling TIF1γ in Non-small Cell Lung Cancer. Mol. Cancer 17 (1), 140. 10.1186/s12943-018-0889-7 30261900 PMC6161470

[B41] Xian J. Su W. Liu L. Rao B. Lin M. Feng Y. (2020). Identification of Three Circular RNA Cargoes in Serum Exosomes as Diagnostic Biomarkers of Non-small-cell Lung Cancer in the Chinese Population. J. Mol. Diagn. 22 (8), 1096–1108. 10.1016/j.jmoldx.2020.05.011 32535085

[B42] Xu Y. Kong S. Qin X. Ju S. (2020). Comprehensive Assessment of Plasma Circ_0004771 as a Novel Diagnostic and Dynamic Monitoring Biomarker in Gastric Cancer. Onco. Targets Ther. 13, 10063–10074. 10.2147/OTT.S263536 33116589 PMC7549879

[B43] Ye R. Tang R. Gan S. Li R. Cheng Y. Guo L. (2020). New Insights into Long Non-coding RNAs in Non-small Cell Lung Cancer. Biomed. Pharmacother. 131, 110775. 10.1016/j.biopha.2020.110775 33152934

[B44] Yu C. Cheng Z. Cui S. Mao X. Li B. Fu Y. (2020). circFOXM1 Promotes Proliferation of Non-small Cell Lung Carcinoma Cells by Acting as a ceRNA to Upregulate FAM83D. J. Exp. Clin. Cancer Res. 39 (1), 55. 10.1186/s13046-020-01555-5 32228656 PMC7106704

[B45] Zaphiropoulos P. G. (1997). Exon Skipping and Circular RNA Formation in Transcripts of the Human Cytochrome P-450 2C18 Gene in Epidermis and of the Rat Androgen Binding Protein Gene in Testis. Mol. Cel Biol. 17 (6), 2985–2993. 10.1128/MCB.17.6.2985 PMC232150 9154796

[B46] Zhang Y. Zhang X.-O. Chen T. Xiang J.-F. Yin Q.-F. Xing Y.-H. (2013). Circular Intronic Long Noncoding RNAs. Mol. Cel 51 (6), 792–806. 10.1016/j.molcel.2013.08.017 24035497

[B47] Zhang P.-F. Pei X. Li K.-S. Jin L.-N. Wang F. Wu J. (2019). Circular RNA circFGFR1 Promotes Progression and Anti-PD-1 Resistance by Sponging miR-381-3p in Non-small Cell Lung Cancer Cells. Mol. Cancer 18 (1), 179. 10.1186/s12943-019-1111-2 31815619 PMC6900862

[B48] Zhang J. Mao W. Chen Z. Gu H. Lian C. (2020). Clinical Significance of Has_circ_0060937 in Bone Metastasis of NSCLC. Int. J. Gen. Med. 13, 1115–1121. 10.2147/IJGM.S279023 33209054 PMC7670089

[B49] Zhao J. Lee E. E. Kim J. Yang R. Chamseddin B. Ni C. (2019). Transforming Activity of an Oncoprotein-Encoding Circular RNA from Human Papillomavirus. Nat. Commun. 10 (1), 2300. 10.1038/s41467-019-10246-5 31127091 PMC6534539

[B50] Zheng Y.-W. Li R.-M. Zhang X.-W. Ren X.-B. (2013). Current Adoptive Immunotherapy in Non-small Cell Lung Cancer and Potential Influence of Therapy Outcome. Cancer Invest. 31 (3), 197–205. 10.3109/07357907.2013.775294 23477587

[B51] Zhou X. Liu H. Y. Wang W. Y. Zhao H. Wang T. (2018). Hsa_circ_0102533 Serves as a Blood-Based Biomarker for Non-small-cell Lung Cancer Diagnosis and Regulates Apoptosis *In Vitro* . Int. J. Clin. Exp. Pathol. 11 (9), 4395–4404. 31949836 PMC6962954

[B52] Zhu X. Wang X. Wei S. Chen Y. Chen Y. Fan X. (2017). hsa_circ_0013958: a Circular RNA and Potential Novel Biomarker for Lung Adenocarcinoma. FEBS J. 284 (14), 2170–2182. 10.1111/febs.14132 28685964

[B53] Zukotynski K. A. Hasan O. K. Lubanovic M. Gerbaudo V. H. (2021). Update on Molecular Imaging and Precision Medicine in Lung Cancer. Radiol. Clin. North Am. 59 (5), 693–703. 10.1016/j.rcl.2021.05.002 34392913

