# Supplementary material for: Comprehensive Assessment of Serum hsa_circ_0070354 as a Novel Diagnostic and Predictive Biomarker in Non-small Cell Lung Cancer
Source: Front Genet. 2022 Jan 13;12:796776. doi: 10.3389/fgene.2021.796776 (PMC8793632; doi:10.3389/fgene.2021.796776)
Supplement: Supplementary file 1 [file Table1.DOCX]

**Supplementary Table 1** Basic information of tissue samples from NSCLC patients.

| No. | Gender | Age | Smoking status | Pathological diagnosis | TNM stage |
| --- | --- | --- | --- | --- | --- |
| 1 | M | 63 | 40Y*15/d | adenocarcinoma | pT2N0M0 |
| 2 | M | 76 | 50Y*20/d | adenocarcinoma | pT2N1M0 |
| 3 | F | 76 | None | adenocarcinoma | pT1N0M0 |
| 4 | M | 72 | 50*20/d | adenocarcinoma | pT3N0M0 |
| 5 | M | 55 | None | squamous cell carcinoma | pT1N0M0 |
| 6 | F | 70 | None | adenocarcinoma | pT2bN0M0 |
| 7 | M | 73 | None | squamous cell carcinoma | pT2bN2M0 |
| 8 | M | 71 | 30Y*20/d, smoking cessation for 20 years | squamous cell carcinoma | pT2N2M0 |
| 9 | M | 73 | None | adenocarcinoma | pT2aN1M0 |
| 10 | M | 69 | 30Y*20/d, smoking cessation for 3 years | adenocarcinoma | pT2N0M0 |
| 11 | F | 69 | None | adenocarcinoma | pT3N2M0 |
| 12 | M | 58 | None | adenocarcinoma | pT1cN2M0 |
| 13 | F | 62 | None | adenocarcinoma | pT2N0M0 |
| 14 | F | 47 | None | adenocarcinoma | pT1N0M0 |
| 15 | F | 55 | None | adenocarcinoma | pT1N0M0 |
| 16 | F | 31 | None | squamous cell carcinoma | pT3N0M0 |

* The samples of No.3-5 were subjected to the transcriptome sequencing.
