# Supplementary figures and images for: Comprehensive Assessment of Serum hsa_circ_0070354 as a Novel Diagnostic and Predictive Biomarker in Non-small Cell Lung Cancer
Source: Front Genet. 2022 Jan 13;12:796776. doi: 10.3389/fgene.2021.796776 (PMC8793632; doi:10.3389/fgene.2021.796776)

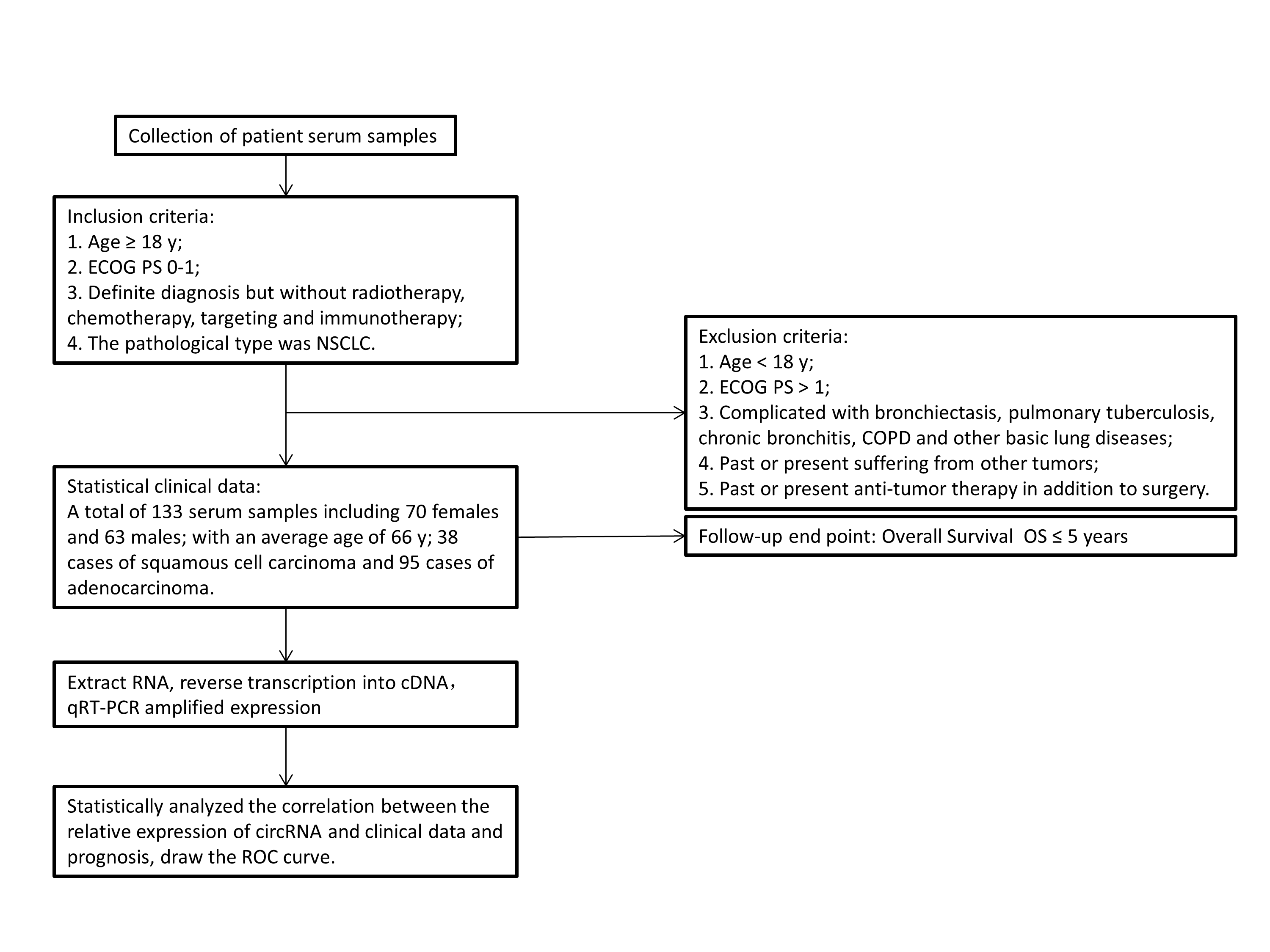

Supplement: Supplementary file 2 [file Image1.TIF]
